# A point-like thermal light source as a probe for sensing light-matter interaction

**DOI:** 10.1038/s41598-022-07668-5

**Published:** 2022-03-22

**Authors:** S. Korn, M. A. Popp, H. B. Weber

**Affiliations:** Lehrstuhl Für Angewandte Physik, Friedrich-Alexander-Universität Erlangen-Nürnberg, 91058 Erlangen, Germany

**Keywords:** Nanophotonics and plasmonics, Microresonators

## Abstract

Historically, thermal radiation is related to 3D cavities. In practice, however, it is known that almost any hot surface radiates according to Planck’s law. This approximate universality roots in the smooth electromagnetic mode structure of free space, into which the radiation is emitted. Here, we study the effect for a strongly patterned mode structure and use quasi-transparent point-like thermal light emitters as a probe. As such, we choose current-driven graphene nanojunctions for which the emission into free space obeys Planck’s law. Placed in front of a mirror, however, this process is highly sensitive to a node/antinode pattern of light modes. By varying the distance, we can sample the latter with atomic precision, and observe a deep imprint on the observed spectrum. The experiment allows an unprecedented view on thermal radiation in a spatially/spectrally patterned electromagnetic environment.

## Introduction

Cavity quantum electrodynamics (cavity QED) is the description of light-matter interaction within quantized light fields in a confined space. A fundamental example is the control of light emission of an atom in between two mirrors (i.e. a reflecting cavity)^1^. The presence of the cavity imposes significant modifications also to materials^2^ and molecules^3^, even in the absence of light. The first appearance of the quantum nature of light fields, however, is Planck’s explanation^4^ of blackbody radiation. This fundamental mechanism, sometimes termed cavity radiation, assumes fully absorbing cavities. Nonetheless, the Planck formula for thermal radiation describes the interaction of nearly any material surface with the light field. This only seemingly universal phenomenology is related to the quasi-smooth mode structure of free space present in most experiments. In recent years, however, various theoretic and experimental investigations have been performed on deviations from thermal Planck spectra caused by geometric patterning on the wavelength-scale^5–9^. We address the interplay of Planck spectra and electromagnetic mode structure experimentally.

In experimental analogy to the theoretical concept of Green’s functions, we consider a point-like broadband source as an ideal choice for sampling light-matter interaction. In contrast to recent experiments with atomically sharp tips like in a scanning tunneling microscope (STM), where the metallic tip is highly invasive, obscures the optical access and modifies the electromagnetic environment drastically^10–12^, we choose an almost non-invasive strategy. Further, we seek for directional ($\vec{k}$) resolution.

## Experimental concept

The experiment is designed such that local mode structures are detectable almost non-invasively. We use a point-like thermal light source and measure the system’s response along a direction of interest. By choosing a broadband source, we can investigate all wavelengths simultaneously. It is provided by an electrically driven graphene nanojunction (GNJ). The graphene layer is atomically thin and nearly transparent. Only 2.3% of the light is absorbed at any wavelength^13^, hence also emissivity is spectrally flat according to Kirchhoff’s law. We use *epitaxial* graphene on 4H silicon carbide (SiC)^14^, which is particularly robust^15^. By lithography and subsequent electroburning, electric nanojunctions can be defined^16^. When a finite current (controlled via an applied voltage $V_{source}$) is driven through, the voltage drop at the nanojunction along with electron–electron interaction provides a hot-electron spot. Its electronic temperatures reach values as high as $T_{Planck} = 3000\,{\text{K}}$, observed as almost perfect Planck spectra when radiating into an open half-space^17^. By modeling the heat spread within the graphene sheet, the size of the spot is estimated to be less than micrometers laterally. Indeed, it appears optically below the resolution limit. In normal direction an atomically sharp confinement can be assumed, because hot electrons are confined within the graphene plane^17^.

**Figure 1:**
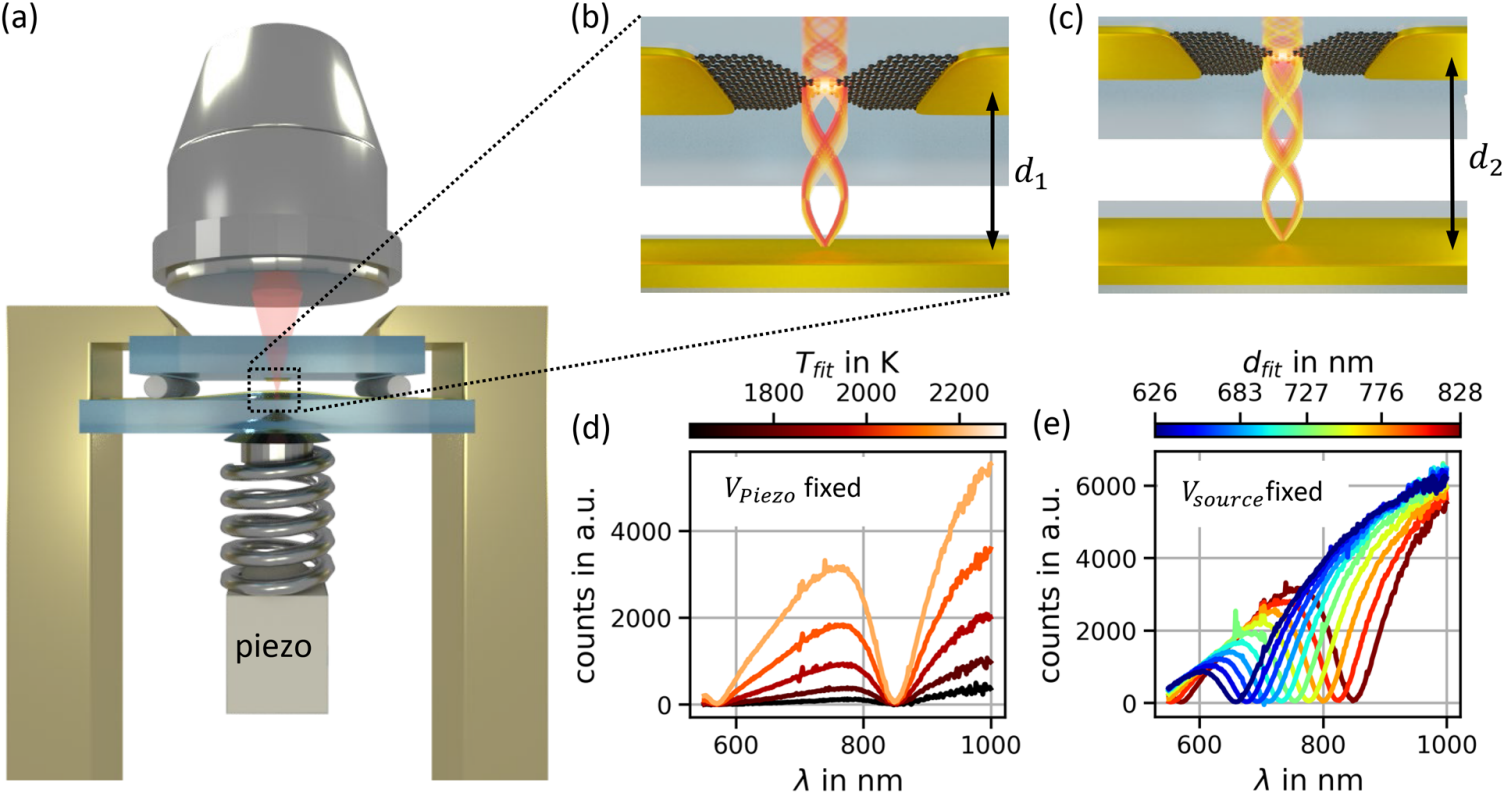
(**a**) Sqeezable nanojunction setup. (**b**) Graphene nanojunction placed in front of a gold mirror with standing wave modes of different wavelength. (**c**) Same as (**b**) but at increased distance. The electromagnetic node at the GNJ is associated with a higher wavelength. (**d**) Example spectra for varied driving voltage $V_{source}$ (resulting in varied temperatures). (**e**) Example spectra for varied distances (in between the physical situations depicted in (**b**) and (**c**)).

As a simple realization of a spatially patterned mode structure we use the standing waves in front of a metallic (Au) mirror, providing nodes at distance $d_{node} \approx \frac{m\lambda }{2}$ ($\lambda$ is the wavelength, $m$ is an integer) The experiment is performed within a Squeezable Nanojunction setup (SNJ, see Fig. 1a), in which the distance can be controlled via a Piezo voltage $V_{Piezo}$. While in former experiments^18,19^ the SNJ was used as an electronic nanojunction it serves here as a tool to create tunable distances between parallel surfaces. On the top chip we pattern a GNJ device and place it in front of a gold mirror on the bottom chip (see Fig. 1b,c). The distance $d$ is tunable in a range of 0…5 µm. We detect light from the top, i.e. through the SiC chip that is supporting the GNJ. Choosing small numerical apertures (NA = 0.12), we record in a first step light directed perpendicular to the mirror plane.

The underlying physical picture is similar to our previous work^20^ where we investigated excitation and emission coupling factors of photoluminescence. However, it provides a conceptual simplification: by using an electrically driven emitter, the influence of spatially dependent excitation is removed. Such, we can directly quantify the local coupling to electromagnetic modes, as described below.

## Results and discussion

Figure 1d shows experimental spectra with fixed distance $d$. Spectra recorded for different $V_{source}$ are color-coded. The spectrum can be described qualitatively by a broadband spectrum, however with deep imprints at $\lambda_{m = 2,d} \approx 840\,{\text{nm}}$ and $\lambda_{m = 3,d} \approx 580\,{\text{nm}}$ (the notation will be justified below). Increasing the voltage leads to increased intensity and a slight blue shift (similar to thermal spectra), but the suppressions at 580 nm and 840 nm remain. When, in a different subset of measurements, $d$ is modified ($V_{source}$ = const.), the imprints $\lambda_{m = 2,d}$ and $\lambda_{m = 3,d}$ shift proportionally to $d$. The envelope of the total set of curves in Fig. 1e resembles strikingly Planck’s law.

The overall shape is readily interpreted as thermal radiation, similar to the measurements without mirror (see Fig. S1).

We discuss two pictures within which the obvious deviations from Planck spectra can be understood:

1. *Partial rays* Thermal radiation (initially with Planck spectrum) of equal amplitude is sent out in opposite directions. The lower beam is reflected at the mirror and interferes with the upper one. The detector measures the squared amplitude of their superposition. Constructive or destructive interference conditions are met depending on $d$ and $\lambda$, resulting in enhanced or decreased spectral intensity, respectively. This picture, which can be enriched by multiply reflected partial beams, gives an intuitive interpretation of the modification of the Planck spectrum.

Better adapted to our case is, however, the following picture:

(2) *Mode analysis* Standing wave modes are formed in front of the mirror by inversion of the actual beam path (light waves with amplitude $E_{0}$ sent from the detector). The light-matter coupling of the GNJ thermal radiator is proportional to the local intensity ($\left| E \right|^{2}$) of these modes. When the GNJ is positioned in an antinode or node of the $\lambda$-dependent mode pattern, the observed spectral intensity is enhanced or blocked, respectively. This can be expressed by the formula for the effective intensity
  1 $$I_{eff} \left( {\lambda ,d} \right) = I_{Planck} \left( \lambda \right) \cdot \frac{{\left| {E_{\lambda ,d} \left( {\vec{x}_{GNJ} } \right)} \right|^{2} }}{{\left| {E_{0} } \right|^{2} }}$$

with the position of the GNJ $\vec{x}_{GNJ}$. The latter term denotes the modification factor encoding the influence of the mode structure.

The equivalence of the pictures (1) and (2) roots in the Lorentz reciprocity principle: the coupling between two antennae does not depend on the direction of signal transfer. It is valid for almost any physical system and material (as long as time-invariance can be assumed).

**Figure 2:**
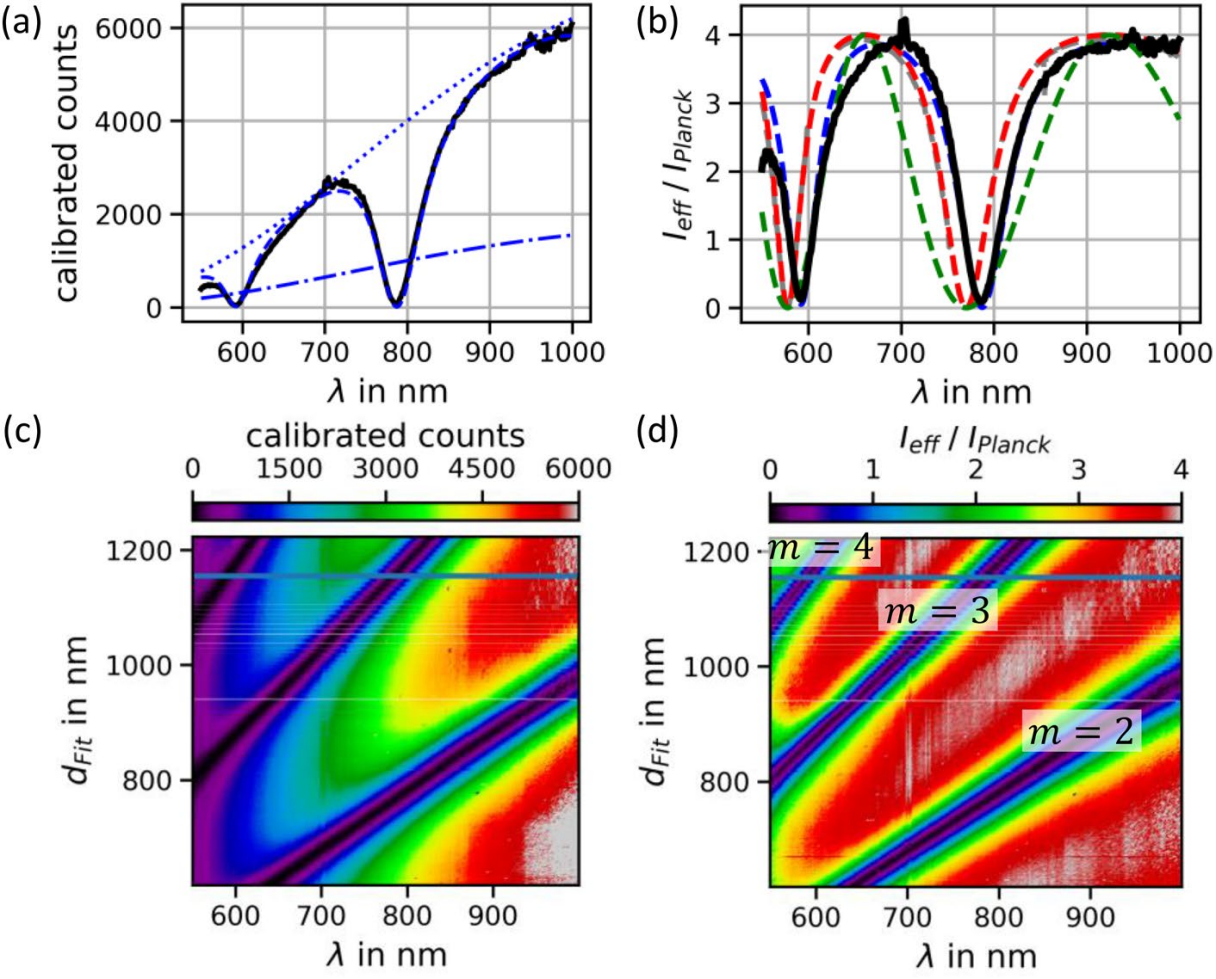
The left column shows calibrated experimental spectra, the right column shows modification factors to Planck part according to Eq. (1). The blue dashed line in (**a**) corresponds to the complete TMM fit model, the dash-dotted line is $I_{Planck}$. The dotted line is $4 \cdot I_{Planck}$ which also serves as envelope. The deep imprints originate from destructive interference, the fourfold enhancement of intensity stems from constructive 2-path interference. The dashed lines in (**b**) showcase the contributions from the dielectric and material specifics of the mirror, see main text. (**c**) Shows the full body of calibrated experimental spectra. (**d**) Displays the modification factor of all data according to Eq. (1).

Technically, we constrict ourself to the 1-dimensional case and evaluate the local fields via the transfer matrix method (TMM)^21^. In our experiment, the two parallel dielectrics provide a Fabry–Perot stack, which should be included in the TMM treatment for quantitative results. In Fig. 2a an example spectrum of $$I_{eff} \left( {\lambda ,d = 1.156\,{\upmu {\rm{m}}}} \right)$$ is shown together with a TMM fit (dashed line) that matches accurately. The TMM model considered here includes the refractive index of the SiC substrate ($$n_{SiC} = 2.6)$$ as well as the wavelength dependent complex refractive indices of the Au mirror and graphene. From this model we can derive fictitious spectra that are displayed in Fig. 2a: the dash-dotted line is $$I_{Planck}$$ in absence of the mirror. When this is multiplied with a factor of 4 (constructive interference of the two beams doubles the electric field and quadruples the intensity), the dotted line results which is the upper envelope of each spectrum, which becomes even more obvious when the distance is parametrically varied at given $$V_{source}$$ (Fig. 1e). Figure 2b shows the $$\lambda$$-dependent modification factor $$\frac{{I_{eff} }}{{I_{Planck} }}$$ that is obtained by dividing experimental spectra by the Planck-part of the model fit. In order to connect curve features to model assumptions, we use the distance $$d$$ extracted from the TMM model and apply physical pictures under varying assumptions: the simplest picture (green dashed line) is the GNJ in front of a perfect mirror ($$n_{Au} \to \infty$$) in absence of dielectrics ($$n_{SiC} \to 1$$). Interference between direct and reflected beam result in sinusoidal oscillation proportional to $$\sin^{2} \left( {2\pi d/\lambda } \right)$$. Including the refractive index $$n_{SiC} \left( \lambda \right)$$^22,23^ of the GNJ substrate introduces multiple reflections resulting in sharpened dips (red curve). The situation is comparable to a cavity, however, with low Q-factor. Considering additionally the non-ideality of the gold mirror (blue dashed line) with its complex refractive index $$n_{Au} \left( \lambda \right)$$^23,24^, a reflection phase is introduced corresponding to finite penetration depth. This leads to a subtle red-shift. The finite layer-thickness of the gold mirror and absorption inside the graphene sheet^23,25^ were included but play little role.

Having analyzed individual spectra of Fig. 2a we now face to the full body of data, i.e. a distance-varied ensemble of spectra. The resulting intensities are shown color-coded in Fig. 2c. Extracted modification factors are visualized in Fig. 2d. The latter were determined by normalization of measurement data with the Planck part of the fitted TMM model (analogously to (b)). The modification factors have maxima with a value of 4 throughout the entire measurement range, except for wavelengths below 600 nm. This lower end of the spectra may be affected by inaccuracies during the calibration of our setup: the calibration data were taken using a black-body radiator at $T \approx 900\,^{ \circ } {\text{C}}$ which has little intensity in this wavelength range (see also^17^).

Spectral dips are visible as diagonal dark lines which extrapolate to ($\lambda = 0,d \approx 0$). The inaccuracy in $d$ originates in the above mentioned red-shift due to finite penetration depth of the mirror. The index $m$ corresponds to the GNJ being positioned at the $m$th node of the mode with wavelength $\lambda$. $d_{fit}$ is extracted from the very same model analysis of experimental data. The straight shape of the black lines provides confidence that our model is consistently valid for a large parameter range. Note that the signal vanishes at the dips within our experimental accuracy. This confirms our expectation that the thickness of our emitters is far below the length scale of the mirror modes i.e. $\frac{\lambda }{2}$. Remarkably, within the partial ray picture, the emission towards the mirror and towards the detector completely interfere such that no signal remains in the direction perpendicular to the mirror. This full interference indicates that thermal emission from graphene creates fully coherent light signals in both perpendicular directions.

**Figure 3:**
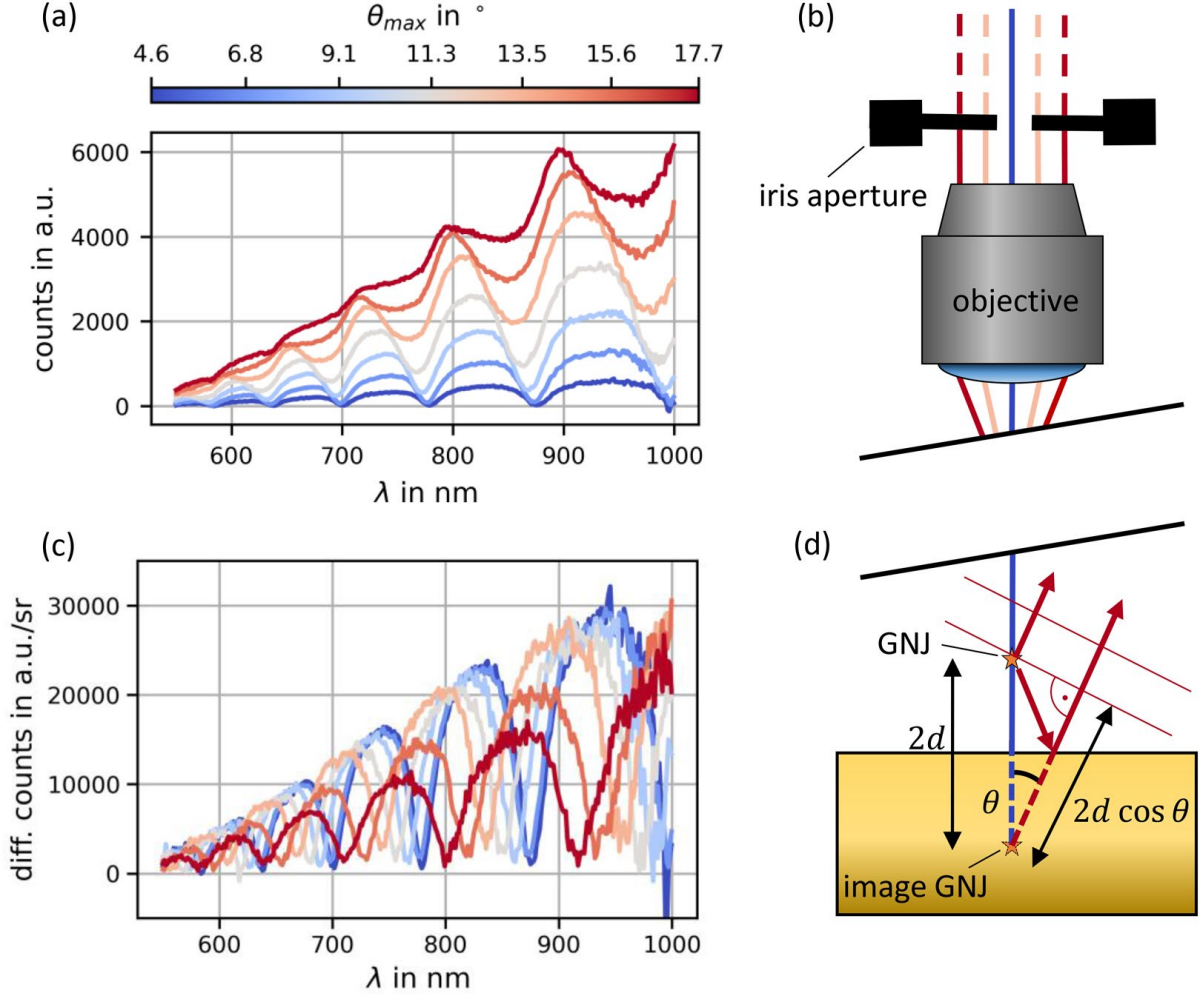
Dependence on numerical aperture. (**a**) Measured intensities at varied maximum beam angle $\theta_{max}$. (**b**) Sketch of the corresponding measurement situation. (**c**) Differential intensity per solid angle. (**d**) Sketch of $\theta$ dependent path difference between direct and reflected ray.

So far, the experiment and our discussion was restricted to a simple 1D-picture. Experimentally this was achieved by blocking off-axis light with an iris aperture (Fig. 3b, beam angles with respect to the optical axis $\theta < \theta_{max}$, $NA = {\text{sin}}\theta_{max}$). In a next step we lift this simplification and target the angular dependence of the effect by varying the diameter of the iris aperture. In addition, we increase the sensitivity to angle dependent phenomena by choosing a larger distance $(d \approx 3.5\,\upmu {\text{m}})$. The higher $d$ causes a smaller spectral spacing between the dips. Figure 3a shows spectra for varied opening angle $\theta_{max}$. For small $\theta_{max}$ destructive interference is still fully observed i.e. the dips approach zero intensity. When increasing the opening angle the total intensity increases and the spectral imprint is weakened.

The measured quantity is an integral over the intensities of all opening angles: $I_{meas} \left( {\theta_{max} } \right) = \mathop \smallint \limits_{0}^{{\theta_{max} }} I\left( \theta \right) \cdot 2\pi \cdot {\text{sin}}\left( \theta \right){\text{d}}\theta$. In order to retrieve $I\left( \theta \right)$ we plot the intensity differentially: $I\left( {\theta_{n} } \right) = \frac{{I_{meas} \left( {\theta_{n} } \right)}}{{\Omega_{n} }} - \frac{{I_{meas} \left( {\theta_{n - 1} } \right)}}{{\Omega_{n - 1} }}$ with solid angle $\Omega = 2\pi \left( {1 - \cos \left( \theta \right)} \right)$, i.e. we decompose the signal into ring-like emission cones. The resulting dataset is displayed in Fig. 3c: The destructive interference pattern is reestablished. For explaining this phenomenon we chose the partial ray picture for a selected cone with opening angle $\theta$: according to the sketch in Fig. 3d the path difference between direct and reflected beam is $2d\cos \theta$, which results in a blue-shift for increasing $\theta$.

While we performed the experiment in front of a mirror, the measurement concept would in principle be capable of resolving the light-matter coupling factor in many other geometries. We see a way to sample local light-matter interaction in the vicinity of micro/nanostructured objects^26,27^ both spatially and directionally.

## Comparison with other experiments

The experiment is related to Drexhage’s seminal experiment^28^, which studies the radiative decay time $\tau_{rad}$ of molecules in front of a mirror. There, it was demonstrated that $\tau_{rad}$ is sensitive to the local electromagnetic density of states, being oscillatory with distance. In particular, at nodes of the standing electromagnetic waves, emission was suppressed in selected directions. Remarkably, also the overall decay time, which is sensitive to the direction-integrated coupling factors, is oscillatory.

Our experiment has a similar geometry, but provides two differences: the light source is (1) broadband, and (2) point-like. As a consequence, we can sample the local mode pattern simultaneously at all wavelengths, and the point-like source will allow for locally resolved sampling.

The question may arise, whether our total, angle-integrated emission is oscillatory with distance like in Drexhage’s case, which would presumably influence the steady-state temperature of the junction. According to our estimates, however, this effect is masked by the dominating non-radiative cooling via electronic coupling within the graphene film and decay to phonons^17^.

Another related class of experiments scans point-like emitters over specimen and measures their decay time^29^. These experiments are sensitive to direction-integrated electromagnetic couplings, while our experiment provides $\vec{k}$-resolution.

## Summary and outlook

We have presented a study using an atomically thin thermal light emitter interacting with a tunable electromagnetic environment. Semi-transparent point-like graphene nanojunctions turn out to be an ideal choice for this purpose. A metallic mirror with tunable distance to the light-source provided an electromagnetic mode pattern. We observed strong modifications of thermal spectra and could assign them as geometry dependent variation of the local far-field coupling. The experiments illustrate how much the electromagnetic mode structure shapes thermal spectra, with the Planck-spectrum as only apparently universal limit. We foresee an application of the method for spatially resolving light-matter coupling in the vicinity of a variety of (nano) objects like metal-tips, plasmonic nano-particles and many others.

## Methods

*SNJ setup* The SNJ setup is composed of two silicon carbide (SiC) chips which are placed face to face. Here, on the top chip graphene nanojunctions (GNJ, see below) are patterned. On the bottom chip large gold areas are patterned, acting as mirrors.

In the uncompressed state the chip surfaces have distances on the order of 5 µm. By squeezing the chips together with a piezo/spring mechanism the distance can be controlled on the sub-nm scale^18^. The SNJ setup is mounted in a vacuum chamber with high vacuum in order to prevent oxidation of the GNJs during operation. Measurements were performed at $T = 150\,{\text{K}}$. At this temperature the stability of the GNJs is drastically increased. Lower temperatures were avoided in order to ensure long measurement times (lower consumption of cryogenics). Recording a full spectrum took ~ 1 min per $V_{source}$ and $d$ value pair, leading to overall measurement times of ~ 1 day.

A detailed description of the SNJ setup can be found in the SI of^20^.

*GNJ chip fabrication* Graphene was grown on semi-insulating 4H–SiC (Cree) by thermal decomposition^14^. The chip thickness is 0.5 mm each. Mechanical contact areas were structured by means of CF_4_ plasma etching in order to keep surface irregularities from generating random mechanical contact points. Each of the contact areas was separated 1.5 mm from the central mesa that was structured with graphene on top. Subsequently gold leads for electrically contacting the central graphene mesa were patterned by means of UV-lithography and thermal evaporation. In a last (e-beam) lithography step and subsequent O_2_-plasma etching constrictions were formed in the graphene sheet. Finally, an electroburning process at ambient conditions was used to create the GNJs^16,30^: Voltage ramps were applied in ambient atmosphere until the conductance dropped below 95% of the maximum value during a ramp. Ramping was repeated until the desired resistance of $R_{0} + \frac{1}{{G_{0} }}$ is reached ($R_{0}$: resistance before burning process, $G_{0} = 2e^{2} /h = 1/12.9\,{{k\Omega }}$: conductance quantum^31^). This resistance regime provides for the remaining electrical connection (1) localization to single/few atoms, (2) still enough electrical power resulting in thermally radiated power, (3) stability for hours of measurements. ^17^.

*Chip preparation mirror chip* The chip carrying the Au mirror was fabricated similarly, however, the graphene growth and O_2_ etching steps were omitted. The Au layer in the relevant central mesa area has a thickness of 80 nm on top of 5 nm adhesive Ti.

*Optical Setup* The optical setup is a custom-built confocal microscope with excitation capabilities including incandescent light (for orientation) and 532 nm laser (not used here). Light is collected by a LCPLFLN20xLCD Olympus objective with correction collar ($NA \approx 0.45$, further restricted by an iris aperture to $NA_{eff} \approx 0.12$ for collection of normally emitted light). No pinhole was used in order to prevent distortion of spectra due to chromatic and birefringent aberrations inside the top SiC chip. An Andor Shamrock 500i Spectrometer with Andor Newton 920 CCD was used for spectroscopy. The wavelength-dependent sensitivity of the whole setup was calibrated using a home-built black body radiation source^17^. For increased $\lambda$ range, spectra at two different center wavelengths (700 nm; 900 nm) were taken for every $d$, accompanied by corresponding background spectra. After background subtraction scaling factors were applied such that the intensity difference in the overlap was minimized, followed by stitching of spectra.

*Electrical* Voltages were applied by an Agilent B2912A precision source measure unit.

*Transfer matrix model (TMM)* A basic description can be found in^21^. Additionally, python code for symbolic formula evaluation was implemented, resulting in speed-up of calculations.

## Supplementary Information

Supplementary Information.

## Data Availability

A file containing raw data, evaluation and visualization code is available under https://doi.org/10.22000/539.
