# Supplementary material for: A point-like thermal light source as a probe for sensing light-matter interaction
Source: Sci Rep. 2022 Mar 22;12:4881. doi: 10.1038/s41598-022-07668-5 (PMC8941087; doi:10.1038/s41598-022-07668-5)
Supplement: Supplementary file 1 — Supplementary Information. [file 41598_2022_7668_MOESM1_ESM.pdf]

A Point like Thermal Light Source as a Probe for Sensing Light-Matter Interaction

# - Supplementary Information -

S.Korn[1†], M. A. Popp[1†], H.B. Weber[1*]

[1] Department of Physics, Lehrstuhl für Angewandte Physik, Friedrich-Alexander-Universität Erlangen-Nürnberg, D- 91058 Erlangen, Germany.

[†] Both authors contributed equally to this manuscript.

[*]corresponding author

Emission from a point-like thermal light source in a non-resonant environment

For comparison, Fig. S1 contains spectral data for a GNJ with varied source voltage V_{source} in absence of a mirror/structured electromagnetic environment. In comparison to Fig. 1 (d) of the main manuscript no spectral imprints are present here. The results are very similar to [1].

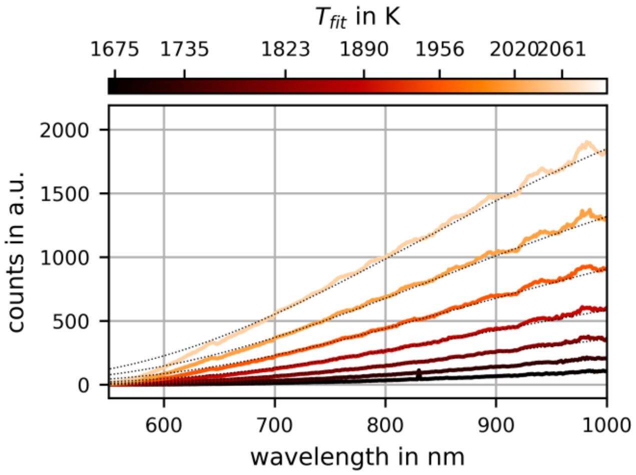

Figure S1. GNJ thermal radiation spectra with varied V_{source} recorded in the absence of a mirror. Dotted lines are fits of bare Planck spectra.

Relation to Drexhage experiment

Fig. S2 shows a further representation of the data shown in Fig. 2 (c) of the main manuscript in order to facilitate comparison with Drexhage's seminal experiment [2]. Our results are related to those of Drexhage but their interpretation should not be confused: Wheras Drexhage analyzes decay times that depend on (reciprocal) angle-integrated coupling factors (DOS) we focus here on coupling

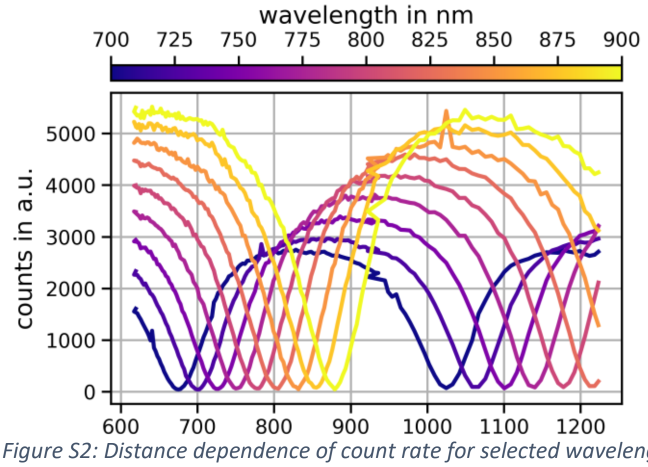

Figure S2: Distance dependence of count rate for selected wavelengths. Same dataset as Fig. 2 (c).

factors to specific modes (with specific direction). Therefore, the features in our data are more pronounced.

Since in our experiment the GNJ was driven in steady state we have no access to decay times. A quantity that should in principle depend on the sum of all decay channels is the temperature T of the thermal radiator. Retrieving T by model fits shows indeed fluctuations on the order of $\pm$100 K, as shown in Fig. S2. It has to be pointed out, however, that these data have to be interpreted with caution: Even slight imperfections in the reflection model (e.g. caused by roughness of the gold surface, deviations in refractive index) can distort the fit function shape, resulting in crosstalk between dip position and fitted temperature. This artifact is especially pronounced when a dip is located at edges of the measurement range. We expect most of the radiated power to be emitted at wavelengths above our measurement range. If there was a physical temperature change we would expect it to be not aligned to spectral features entering our rather arbitrary measurement range which is however the case here.

Further, a previous study [1] has shown that thermal de-excitation of GNJ emitters is dominated by heat conduction inside the electronic system towards a low-temperature thermal bath rather than by photon emission.

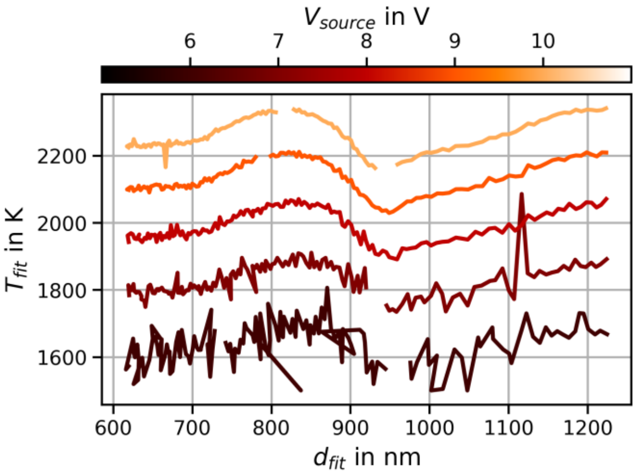

Figure S3: Temperatures retrieved from model fits for varied V_{source} and d. Obvious fit errors were sorted out for clarity.

Bibliography

1. Ott, C., Götzinger, S. & Weber, H. B. Thermal origin of light emission in nonresonant and resonant nanojunctions. *Phys. Rev. Res.* **2**, 042019 (2020).

2. Drexhage, K. H. Influence of a dielectric interface on fluorescence decay time. *J. Lumin.* **1**–**2**, 693–701 (1970).
